# Supplementary material for: User Perceptions of ROTEM-Guided Haemostatic Resuscitation: A Mixed Qualitative–Quantitative Study
Source: Bioengineering (Basel). 2023 Mar 21;10(3):386. doi: 10.3390/bioengineering10030386 (PMC10044818; doi:10.3390/bioengineering10030386)
Supplement: Supplementary file 1 [file bioengineering-10-00386-s001.zip › S1.pdf]

Translated field notes collected during interviews about ROTEM

Participant 1

POSITIVE: What did you LIKE about the ROTEM?

[Values (exact values).]

NEGATIVE: What did you *DISLIKE* about the ROTEM?

[Takes a long time to start after inserting into ROTEM Sigma (10 minutes).] [Has it worked?]

Participant 2

POSITIVE: What did you <u>LIKE</u> about the ROTEM?

[Can read out a lot, including details.] [Can draw a conclusion quickly.] [The progression is visible over time, which would not be visible with the VC?] [Does not take five minutes to interpret, can save time.]

NEGATIVE: What did you *DISLIKE* about the ROTEM?

[Needs some basic knowledge]. [Needs flowchart. How to interpret it.]

Participant 3

POSITIVE: What did you LIKE about the ROTEM?

[Fast availability.]

NEGATIVE: What did you *DISLIKE* about the ROTEM?

[Does not capture many things such as platelets or anticoagulation.]

Participant 4

POSITIVE: What did you LIKE about the ROTEM?

[Exact quantitative values.] [ROTEM is more elaborate.] [Faster there.] [You can see certain things that you do not see in the lab (hyperfibrinolysis).] [Trend over the time.] [Special situations particularly well visible, for example liver insufficiency. Lab then has poor values, but ROTEM can be good.]

NEGATIVE: What did you *DISLIKE* about the ROTEM?

[Xarelto is not detectable.]

Participant 5

POSITIVE: What did you LIKE about the ROTEM?

[More precise quantitative data.]

NEGATIVE: What did you *DISLIKE* about the ROTEM?

[Must be learned extensively.] [Uncertainties exist, so that almost every time you use it, you have to read something from the internet.] [No information regarding platelets.]

Participant 6

POSITIVE: What did you LIKE about the ROTEM?

[Simplifies work because it gives a quick result and does not have to be sent to the lab.] [If you can evaluate it, it is very helpful and efficient in the acute situation.]

NEGATIVE: What did you *DISLIKE* about the ROTEM?

[Must be learned several times a week to be able to interpret it correctly.] [If it is learned regularly, then after a few years you can probably interpret it correctly.]

***Participant** 7*

POSITIVE: What did you LIKE about the ROTEM?

[Fast results available.] [Close to the patient, point-of-care test.]

NEGATIVE: What did you *DISLIKE* about the ROTEM?

[Relatively long duration for consideration, although it is used in critical situations.]
[Diagnostics takes longer] [especially for the inexperienced.]

Participant 8

POSITIVE: What did you <u>LIKE</u> about the ROTEM?

[You know it] [you are used to it] [important things/images known how it should look] [years of experience how it should be.]

NEGATIVE: What did you *DISLIKE* about the ROTEM?

[Too many numbers] [too much red] [no longer know what is important and unimportant.]

Participant 9

POSITIVE: What did you LIKE about the ROTEM?

[Point-of-care, gives direct information about the clotting situation.] [Relatively simple action opportunities depending on what is represented.]

NEGATIVE: What did you *DISLIKE* about the ROTEM?

[Display not user-friendly] [much training required.] [Limitation of imaging because not all clotting problems are detected.]

Participant 10

POSITIVE: What did you LIKE about the ROTEM?

[New ROTEM Sigma is easy to handle, no pipetting required.]

NEGATIVE: What did you *DISLIKE* about the ROTEM?

[It takes experience.] [You have to read up on it.] [You have to deal with it a lot.]

Participant 11

POSITIVE: What did you LIKE about the ROTEM?

[Faster is ROTEM Sigma vs. Delta, easy handling.] [Not quite simple but it gets simple sooner.]

NEGATIVE: What did you *DISLIKE* about the ROTEM?

[Interpretation is difficult.] [Exact detailed knowledge is difficult.]

Participant 12

POSITIVE: What did you LIKE about the ROTEM?

[The duration/progression is visible.]

NEGATIVE: What did you *DISLIKE* about the ROTEM?

[Needs practice] [look long enough on it] [recognize numbers.] [It is unclear if it works or not.]

Participant 13

POSITIVE: What did you LIKE about the ROTEM?

[Despite many values, recognize at a glance whether it is regular or not] [visual aspect thereby.]

NEGATIVE: What did you *DISLIKE* about the ROTEM?

[Confusing] [too much.]

Participant 14

POSITIVE: What did you LIKE about the ROTEM?

[Very good also for cardiac anesthesia] [you see directly the situation.] [It is known and one is used to it.]

NEGATIVE: What did you *DISLIKE* about the ROTEM?

[Takes some time] [you must wait even in emergency situations] [one is therefore “under power”.]

Participant 15

POSITIVE: What did you <u>LIKE</u> about the ROTEM?

[Most important coagulation information on it.]

NEGATIVE: What did you *DISLIKE* about the ROTEM?

[Complicated to interpret.]

Participant 16

POSITIVE: What did you LIKE about the ROTEM?

[Do not know ROTEM yet.]

NEGATIVE: What did you *DISLIKE* about the ROTEM?

[No device available in N2C.] [Find it complicated.] [You have to do it often to interpret it.]

Participant 17

POSITIVE: What did you LIKE about the ROTEM?

[Graphics easy to interpret, good visualization.]

NEGATIVE: What did you *DISLIKE* about the ROTEM?

[Takes a long time to be available.] [Numbers are too small, especially under stress and on night duty.]

Participant 18

POSITIVE: What did you LIKE about the ROTEM?

[A normal coagulation situation or e.g. fibrinogen deficiency is detected directly.]

NEGATIVE: What did you *DISLIKE* about the ROTEM?

[All of the other coagulation disorders can only be detected with a lot of experience.] [The structure of the ROTEM must be known to understand it.] [With a lack of knowledge, the ROTEM cannot be interpreted.]

Participant 19

POSITIVE: What did you <u>LIKE</u> about the ROTEM?

[Visual analysis (with color and shape).] [Quickly available, does not have to be brought to the lab.] [Only one tube is used for it.]

NEGATIVE: What did you *DISLIKE* about the ROTEM?

[Lack of written information on the visual image (it would be helpful if, for example, it said "fibrinogen below 7 = fibrinogen deficiency" or "thrombocytopenia". Written information could still be included and would simplify interpretation.]

Participant 20

POSITIVE: What did you LIKE about the ROTEM?

[Timeline to estimate extent.]

NEGATIVE: What did you *DISLIKE* about the ROTEM?

[Needs training] [is not intuitive.]

Participant 21

POSITIVE: What did you LIKE about the ROTEM?

[Quick information.] [Visual representation (not only numbers).]

NEGATIVE: What did you *DISLIKE* about the ROTEM?

[Complex.] [Limited availability (you have to bring it somewhere else).] [It takes time.] [Result not always visible at the workplace.]

Participant 22

POSITIVE: What did you LIKE about the ROTEM?

[It is in real time] [information is getting continuously more] [you can react early compared to the lab.]

NEGATIVE: What did you *DISLIKE* about the ROTEM?

[Often read through] [trying to learn, have to repeat again and again] [not intuitive.]

Participant 23

POSITIVE: What did you LIKE about the ROTEM?

[ROTEM is much easier since ROTEM Sigma, less error-prone from operability.]

NEGATIVE: What did you *DISLIKE* about the ROTEM?

[Before more error-prone from operation.] [Very sensitive device, so that there are still disturbances.] [Even if you take a wrong tube there are problems, for example those with less blood volume.]

Participant 24

POSITIVE: What did you LIKE about the ROTEM?

[Quantifying what is missing and to what extent.]

NEGATIVE: What did you *DISLIKE* about the ROTEM?

[Nothing.] [If necessary time until the clot is formed, but that is the way it is with clotting.] [Not so easy for beginners.]

Participant 25

POSITIVE: What did you LIKE about the ROTEM?

[Very differentiated (if you can do it).]

NEGATIVE: What did you *DISLIKE* about the ROTEM?

[Not intuitive] [you need a lot of training.]

Participant 26

POSITIVE: What did you LIKE about the ROTEM?

[Time-dependent factor of coagulation (after 5, 10, 20 minutes).]

NEGATIVE: What did you *DISLIKE* about the ROTEM?

- [Many values that are not evaluated (A10, …)] [too much information that is not evaluated (especially in acute cases).]

Participant 27

POSITIVE: What did you <u>LIKE</u> about the ROTEM?

[Important results.] [Quantitative.]

NEGATIVE: What did you *DISLIKE* about the ROTEM?

[Needs experience.]

Participant 28

POSITIVE: What did you LIKE about the ROTEM?

[Chronological sequence with time line, one can see the development.] [Normal ranges available (shown above or below).] [One can quickly see e.g. in the FIBTEM that it will not be good.]

NEGATIVE: What did you *DISLIKE* about the ROTEM?

[Higher concentration needed if you do not use it that often] [you have to “get into it” to know what is not in the normal range (needs to be looked at more closely).]

Participant 29

POSITIVE: What did you LIKE about the ROTEM?

[International (universally recognized).] [Quantitative.]

NEGATIVE: What did you *DISLIKE* about the ROTEM?

[Not clearly arranged.] [Needs a lot of experience.]

Participant 30

POSITIVE: What did you LIKE about the ROTEM?

[Visual.]

NEGATIVE: What did you *DISLIKE* about the ROTEM?

[Needs previous knowledge.]

Participant 31

POSITIVE: What did you LIKE about the ROTEM?

[Time course visible.] [Visualization of coagulation.] [Pathological values displayed in red (-> helps with interpretation).]

NEGATIVE: What did you *DISLIKE* about the ROTEM?

[Needs time before you can see anything.]

Participant 32

POSITIVE: What did you <u>LIKE</u> about the ROTEM?

[Habit, works with it for a long time, you can see it and assess it.] [More concise.]

NEGATIVE: What did you *DISLIKE* about the ROTEM?

[Nothing.]

Participant 33

POSITIVE: What did you <u>LIKE</u> about the ROTEM?

[Visual aspect imprinted.] [Dynamics over time.] [ROTEM already imprinted]

NEGATIVE: What did you *DISLIKE* about the ROTEM?

[Nothing.]

Participant 34

POSITIVE: What did you LIKE about the ROTEM?

[One can estimate how much should be substituted.]

NEGATIVE: What did you *DISLIKE* about the ROTEM?

[Not yet known (only for a short time on the anesthesia).]

Participant 35

POSITIVE: What did you <u>LIKE</u> about the ROTEM?

[Intuitive.] [That there is the possibility (POC).] [Technically further developed and more simple (without pipetting).]

NEGATIVE: What did you *DISLIKE* about the ROTEM?

[Two cartridges negative (technical problem), it is necessary to know if heparin is involved.] [5-10 numbers that make a difference in stress, static.) [In the house: works 50/50 whether it works and is displayed online.]

Participant 36

POSITIVE: What did you LIKE about the ROTEM?

[A lot of information can be obtained in a fast time.]

NEGATIVE: What did you *DISLIKE* about the ROTEM?

[It takes time to master it. As with any technology.]

Participant 37

POSITIVE: What did you LIKE about the ROTEM?

[Graphical representation.] [Has a relatively quick approximate assessment at a glance.]

NEGATIVE: What did you *DISLIKE* about the ROTEM?

[Numbers are shown too small.] [Useful if marked red, if out of normal range.]

Participant 38

POSITIVE: What did you LIKE about the ROTEM?

[More experience, one has always worked with it (for 4 years)] [since exercise it is better now.]

NEGATIVE: What did you *DISLIKE* about the ROTEM?

[At the beginning very difficult to understand it] [not really intuitive.]

Participant 39

POSITIVE: What did you <u>LIKE</u> about the ROTEM?

[Nothing (never interpreted myself).]

NEGATIVE: What did you *DISLIKE* about the ROTEM?

[Too difficult to interpret (also because of numbers).]

Participant 40

POSITIVE: What did you LIKE about the ROTEM?

[Faster now than with pipetting before.] [Safer, because fewer errors, depending on who has done it, it was often not really reliably conclusive in the past.]

NEGATIVE: What did you *DISLIKE* about the ROTEM?

[If it is not accepted, in case of measurement error, the whole tube is automatically gone.][Unclear why it does not work again and again.] [Physical separation between machine and view.] [ROTEM does not work on all PCs e.g. C-section operating room.]

Participant 41

POSITIVE: What did you LIKE about the ROTEM?

[It is faster than the ROTEM Delta. Pipetting was very tedious. Now it is very simple and practical.]

NEGATIVE: What did you *DISLIKE* about the ROTEM?

[Difficult to interpret.][Too many values, if you do not really know it you cannot recognize it immediately, e.g. as a nurse, if you do not use it often.] [There are no alarms or red marking.]

Participant 42

POSITIVE: What did you LIKE about the ROTEM?

[Gaze diagnosis, if experience is already obtained.] [Shows trend.] [Shows values.] [Time factor is displayed (where do you stand in time?).]

NEGATIVE: What did you *DISLIKE* about the ROTEM?

[Needs certain experience] [you have to get used to it.]

Participant 43

POSITIVE: What did you <u>LIKE</u> about the ROTEM?

[Many details and information to extract.]

NEGATIVE: What did you *DISLIKE* about the ROTEM?

[Confusing.] [You have to do it often until you know what information you can extract and where it is written, what something means.]

Participant 44

POSITIVE: What did you LIKE about the ROTEM?

[Categorically divided (intrinsic/extrinsic) – clearly structured.] [With HEPTEM and FIBTEM you can further decide what to substitute.] [Time course better with ROTEM]

NEGATIVE: What did you *DISLIKE* about the ROTEM?

[You have to read again and again what is shown in which figure and which substances are detected, sometimes not trivial to conclude if and what to substitute and how much (e.g. platelets yes/no and how much).]

Participant 45

POSITIVE: What did you LIKE about the ROTEM?

[Handling is easier than before.]

NEGATIVE: What did you *DISLIKE* about the ROTEM?

[Complicated interpretation.]

Participant 46

POSITIVE: What did you LIKE about the ROTEM?

[Nothing.]

NEGATIVE: What did you *DISLIKE* about the ROTEM?

[Difficult interpretation for beginners.]

Participant 47

POSITIVE: What did you LIKE about the ROTEM?

[Coagulation status is detected rather quickly.]

NEGATIVE: What did you *DISLIKE* about the ROTEM?

[A lot of people (manpower) is needed for the analysis.]

Participant 48

POSITIVE: What did you LIKE about the ROTEM?

[That this exists.]

NEGATIVE: What did you *DISLIKE* about the ROTEM?

[User-unfriendly because no teaching experience although background knowledge is available but not specific of the ROTEM.]

Participant 49

POSITIVE: What did you LIKE about the ROTEM?

[If you understand the theory you can figure out a diagnosis with a few looks.]

NEGATIVE: What did you *DISLIKE* about the ROTEM?

[Not very intuitive.] [Many shortcuts, you have to deal with it more often.][Another system to learn, overwhelming at the beginning.] [Too complex.]

Participant 50

POSITIVE: What did you LIKE about the ROTEM?

[Result quite fast.]

NEGATIVE: What did you *DISLIKE* about the ROTEM?

[Visualization difficult.] [Needs more time and routine to interpret it.]

Participant 51

POSITIVE: What did you LIKE about the ROTEM?

[Not too many colors][pattern is not too complex.] [One recognizes it faster also with the help of the numbers] [and is faster in initiating therapy.]

NEGATIVE: What did you *DISLIKE* about the ROTEM?

[Is generally not practiced often] [no training, no posters/algorithm.]

Participant 52

POSITIVE: What did you LIKE about the ROTEM?

[You have numbers] [and see what is low.] [It is more common (e.g. if you change the house and have only worked with VC so far, you would have to get used to ROTEM again).]

NEGATIVE: What did you *DISLIKE* about the ROTEM?

[You have to look at it more often (e.g. hyperfibrinolysis is only seen last.)- Sometimes people forget to look at it later as well.] [Helpful if it would blink when something is wrong (an amateur then would also recognize that something is wrong).]

Participant 53

POSITIVE: What did you LIKE about the ROTEM?

[You can see the quantitative, how much is missing approximately.] [If you have a system to read it, you will quickly get to the right information.]

NEGATIVE: What did you *DISLIKE* about the ROTEM?

[If you do not have so much experience, it takes longer to get into it.]

Participant 54

POSITIVE: What did you LIKE about the ROTEM?

[Absolute values available.] [Standard you are used to.] [Reference point available (how bad is it really?).] [Can be balanced more accurately (4g fibrinogen instead of only 2g)]

NEGATIVE: What did you *DISLIKE* about the ROTEM?

[Interpret more by yourself.]

Participant 55

POSITIVE: What did you LIKE about the ROTEM?

[Well-structured.] [Course visible.]

NEGATIVE: What did you *DISLIKE* about the ROTEM?

[Numbers are small in relation to the image (you have to search long to find/see them).]

Participant 56

POSITIVE: What did you LIKE about the ROTEM?

[Graphical illustration.]

NEGATIVE: What did you *DISLIKE* about the ROTEM?

[Too many values to interpret.]

Participant 57

POSITIVE: What did you LIKE about the ROTEM?

[Clear structure.] [Many information.]

NEGATIVE: What did you *DISLIKE* about the ROTEM?

[Nothing.]

Participant 58

POSITIVE: What did you LIKE about the ROTEM?

[One can better illustrate the individual factors (CT).]

NEGATIVE: What did you *DISLIKE* about the ROTEM?

[No experience with it.]

Participant 59

POSITIVE: What did you LIKE about the ROTEM?

[Exciting case with heparin.]

NEGATIVE: What did you *DISLIKE* about the ROTEM?

- [Nothing.]

Participant 60

POSITIVE: What did you LIKE about the ROTEM?

[Quick to interpret (if you do it often).] [Quite a lot of information.]

NEGATIVE: What did you *DISLIKE* about the ROTEM?

[Needs practice (you forget it quickly if you do not use it often).]

Participant 61

POSITIVE: What did you LIKE about the ROTEM?

[One is more used to it.] [More information, more quantification (numbers).]

NEGATIVE: What did you *DISLIKE* about the ROTEM?

[It takes time to understand it well.]

Participant 62

POSITIVE: What did you LIKE about the ROTEM?

[One has numbers] [it is more detailed] [with experience and if one can read it, one can extract more from it.]

NEGATIVE: What did you *DISLIKE* about the ROTEM?

[Little ROTEM experience, if you do not know it, interpretation is not self-explanatory; even with legend you do not know effectively what the result means.]

Participant 63

POSITIVE: What did you LIKE about the ROTEM?

[Numerical value (reference value).] [Great tool, goes faster now (no more pipetting, less time required).]

NEGATIVE: What did you *DISLIKE* about the ROTEM?

[No tutorial for ROTEM available (how to run a sample).]

Participant 64

POSITIVE: What did you LIKE about the ROTEM?

[You can quickly see everything at a glance.]

NEGATIVE: What did you *DISLIKE* about the ROTEM?

[It takes a moment until you understand how it works and what it means – especially because you have to wait for values.][It takes more effort to understand it (when do I give TXA, fibrinogen, fibrogamine).]

Participant 65

POSITIVE: What did you LIKE about the ROTEM?

[Visual.] [Values displayed.]

NEGATIVE: What did you *DISLIKE* about the ROTEM?

[It is made unnecessarily complicated (design).]

Participant 66

POSITIVE: What did you LIKE about the ROTEM?

[Values available for assessment.]

NEGATIVE: What did you *DISLIKE* about the ROTEM?

[You have to be familiar with the system.]

Participant 67

POSITIVE: What did you LIKE about the ROTEM?

[Familiar technique, you have more practice.] [Numeric values.]

NEGATIVE: What did you *DISLIKE* about the ROTEM?

[Partially complex interpretation.]

Participant 68

POSITIVE: What did you LIKE about the ROTEM?

[Good overview of coagulation disorders.] [Rather fast.]

NEGATIVE: What did you *DISLIKE* about the ROTEM?

[Previous knowledge necessary for the interpretation.]

Participant 69

POSITIVE: What did you LIKE about the ROTEM?

[You can see numbers][and you can see how narrow the gradients are.]

NEGATIVE: What did you *DISLIKE* about the ROTEM?

[With less experience you need more time until you determine a diagnosis.]

Participant 70

POSITIVE: What did you LIKE about the ROTEM?

[Numeric/absolute numbers.] [You can follow the guidelines.] [Good that you no longer have to pipette.]

NEGATIVE: What did you *DISLIKE* about the ROTEM?

[More training (also from the nursing side) needed.][Not well-founded in the education.]
[One must always get out to look at the ROTEM, does not always work on the PC.]

Participant 71

POSITIVE: What did you LIKE about the ROTEM?

[Quantification available (MCF).]

NEGATIVE: What did you *DISLIKE* about the ROTEM?

[Needs experience and practice.]

Participant 72

POSITIVE: What did you LIKE about the ROTEM?

[If you are used to it, you can read out a lot of it (how much fibrinogen from FIBTEM).]

NEGATIVE: What did you *DISLIKE* about the ROTEM?

[Takes a while to get the necessary information from it.] [Needs practice.]

Participant 73

POSITIVE: What did you LIKE about the ROTEM?

[Already known.] [Times specified.]

NEGATIVE: What did you *DISLIKE* about the ROTEM?

[Needs a lot of background knowledge and expertise (education and training).]

Participant 74

POSITIVE: What did you <u>LIKE</u> about the ROTEM?

[Color component and numbers (statement).] [Additional help for lack of factors (in combination with other results).] [POC.]

NEGATIVE: What did you *DISLIKE* about the ROTEM?

[Condition interpretation, duration until it is interpretable.]

Participant 75

POSITIVE: What did you LIKE about the ROTEM?

[Hard facts (values, quantitative).]

NEGATIVE: What did you *DISLIKE* about the ROTEM?

[Needs a lot of practice.]

Participant 76

POSITIVE: What did you LIKE about the ROTEM?

[Absolute numbers available.] [Compact presentation.] [A lot of information about factors.]

NEGATIVE: What did you *DISLIKE* about the ROTEM?

[Does not always correlate with laboratory, not always 100% with surgeon. Coagulation situation (looks good, but surgeon complains anyway).]

***Participant** 77*

POSITIVE: What did you LIKE about the ROTEM?

[Quickly done.] [FIBTEM is decisive, you can also see if hyperfibrinolysis is present.]

[More complicated.] [Must look more at the numbers] [glasses must be put on.]
[EXTEM/INTEM is looked at less.]

Translated with www.DeepL.com/Translator (free version)
