# Supplementary material for: User Perceptions of ROTEM-Guided Haemostatic Resuscitation: A Mixed Qualitative–Quantitative Study
Source: Bioengineering (Basel). 2023 Mar 21;10(3):386. doi: 10.3390/bioengineering10030386 (PMC10044818; doi:10.3390/bioengineering10030386)
Supplement: Supplementary file 1 [file bioengineering-10-00386-s001.zip › S2.pdf]

Translated online survey invitation

Dear colleagues

In order to better understand and take into account your needs when working with rotational thromboelastometry (ROTEM), our research group has created a short survey that reflects your views on various aspects of using it.

Your opinion will help to develop a user-friendly and user-oriented working environment and contribute to further research on this and related topics.

Link to the survey (max. 2 minutes): -

Kind regards, and thank you for helping us make life-saving decisions!

Your research team

Translated with www.DeepL.com/Translator (free version)
